# Laboratory Diagnosis for Outbreak-Prone Infectious Diseases after Typhoon Yolanda (Haiyan), Philippines

**DOI:** 10.1371/currents.dis.9c3cb7b01ec2d04eef2406dbe03d253d

**Published:** 2016-10-21

**Authors:** Mariko Saito-Obata, Mayuko Saito, Titus C. Tan, Inez Andrea P. Medado, Clyde Dapat, Michiko Okamoto, Raita Tamaki, Rowena C. Capistrano, Edelwisa Segubre-Mercado, Socorro P. Lupisan, Hitoshi Oshitani

**Affiliations:** Department of Virology, Tohoku University Graduate School of Medicine, Sendai, Japan; RITM-Tohoku Collaborating Research Center on Emerging and Re-emerging Infectious Diseases, Muntinlupa City, Philippines; Department of Virology, Tohoku University Graduate School of Medicine, Sendai, Japan; RITM-Tohoku Collaborating Research Center on Emerging and Re-emerging Infectious Diseases, Muntinlupa City, Philippines; Research Institute for Tropical Medicine, Muntinlupa City, Philippines; RITM-Tohoku Collaborating Research Center on Emerging and Re-emerging Infectious Diseases, Muntinlupa City, Philippines; Research Institute for Tropical Medicine, Muntinlupa City, Philippines; Department of Virology, Tohoku University Graduate School of Medicine, Sendai, Japan; Department of Virology, Tohoku University Graduate School of Medicine, Sendai, Japan; Department of Virology, Tohoku University Graduate School of Medicine, Sendai, Tohoku, Japan; RITM-Tohoku Collaborating Research Center on Emerging and Re-emerging Infectious Diseases. Muntinlupa City, Philippines; Research Institute for Tropical Medicine, Muntinlupa City, Philippines; Research Institute for Tropical Medicine, Molecular Biology Laboratory, Muntinlupa City, Philippines; Research Institute for Tropical Medicine, Muntinlupa City, Philippines; Department of Virology, Tohoku University Graduate School of Medicine, Sendai, Miyagi, Japan

## Abstract

Introduction: Typhoon Yolanda (Haiyan) hit the central part of the Philippines on November 8, 2013. To identify possible outbreaks of communicable diseases after the typhoon, nasopharyngeal swabs, stool and blood samples were collected from patients who visited the Eastern Visayas Regional Medical Center due to acute respiratory infection (ARI), acute gastroenteritis (AGE) or other febrile illness (OFI) including suspected dengue fever, between November 28, 2013 and February 5, 2014.

Methods: Samples were tested on-site for selected pathogens using rapid diagnostic tests. Confirmation and further analysis were conducted at the Research Institute for Tropical Medicine (RITM) in Manila using polymerase chain reaction (PCR) and sequencing. Residues of the rapid diagnostic tests and samples collected in the filter papers (FTATM card) were transported to Manila under suboptimal conditions. PCR results were compared between the kit residues and the filter papers.

Results: A total of 185 samples were collected. Of these, 128 cases were ARI, 17 cases were AGE and 40 cases were OFI. For nasopharyngeal swab samples, detection rates for enterovirus and rhinovirus residues were higher than the filter papers. For stool samples, rotavirus positive rate for the filter paper was higher than the kit residues. We also managed to obtain the sequence data from some of the kit residues and filter papers.

Discussion: Our results confirmed the importance of PCR for the laboratory diagnosis of infectious diseases in post-disaster situations when  diagnostic options are limited.

## Introduction

Typhoon Yolanda (Haiyan) hit the central part of the Philippines on November 8, 2013, ­­which caused devastating damages including 6,300 reported deaths and 1,062 people missing^1^. After major disasters, outbreaks of communicable diseases may occur due to poor sanitation and crowded conditions 2 ^,^ 3. Furthermore, the risk of communicable disease outbreak is often exaggerated. Therefore, it is important to monitor if there are any outbreaks in affected areas after a disaster. However, laboratory confirmation is usually not available due to lack of electricity, necessary laboratory materials, and system for transporting samples. To date, there are no general guidelines for laboratory testing in post-disaster settings. Thus, in this study, we conducted laboratory diagnosis of communicable diseases after a strong typhoon, where cold chain was not available.

The Tohoku University of Japan and the Research Institute for Tropical Medicine (RITM) of the Philippines established the RITM-Tohoku Collaborating Research Center on Emerging and Re-emerging Infectious Diseases. The center has been conducting researches on childhood pneumonia, influenza-like illness and acute gastroenteritis in Tacloban City and surrounding areas since 2008 (Figure 1) 4 ^,^ 5. The research laboratory that had been established inside the Eastern Visayas Regional Medical Center (EVRMC) compound was totally damaged during the typhoon (Figure 2). We provided laboratory support to conduct laboratory diagnosis for samples collected from patients who visited EVRMC after the typhoon. Various rapid test kits that were selected based on our research data and past surveillance information in the area were provided for on-site diagnosis^6^^,^ 7. In addition, to detect possible outbreaks and other pathogens that were outside the scope of rapid test kits, samples were collected in FTA^TM^ cards (GE Healthcare Lifesciences), which is a commercially available filter paper treated chemically to stabilize nucleic acids even without cold chain^8^. Also, residue samples used for the rapid test kits, were used for PCR and subsequent sequencing analysis.

**Location map of the study site:**
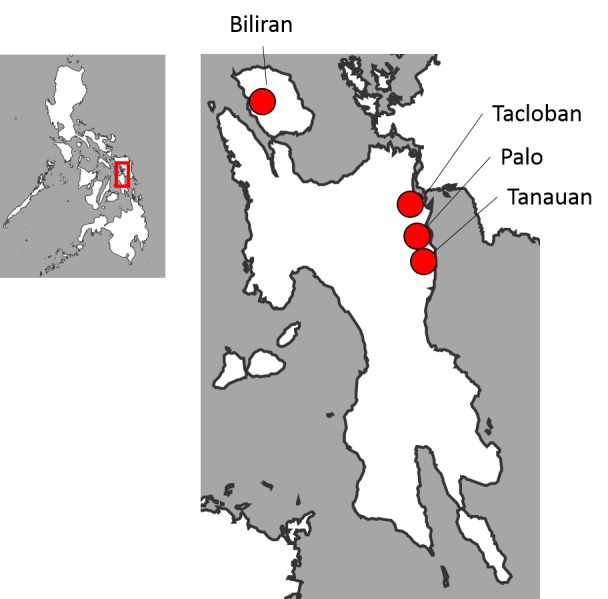

**Wiped-out laboratory building of Tohoku-RITM Collaborating Research Center inside the EVRMC compound:**
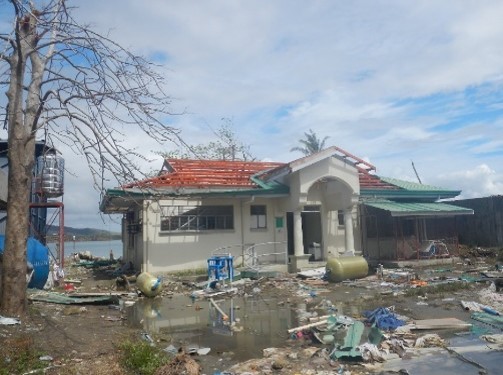
Photo was taken on Nov 24, 2013

## Methods

**Sample collection**

Samples collected from patients who visited EVRMC with acute respiratory infection (ARI), acute gastroenteritis (AGE) or other febrile infections (OFI) from November 28, 2013 to February 5, 2014 were included in the study. Patient’s demographic information was taken and rapid tests were performed based on the main symptoms (Table 1). The residues of nasopharyngeal swabs (NPS) or stool suspension used for the rapid tests were kept for PCR. Specimens taken for the rapid tests were also directly applied to FTA^TM^ cards and then used for PCR.

**Figure:**
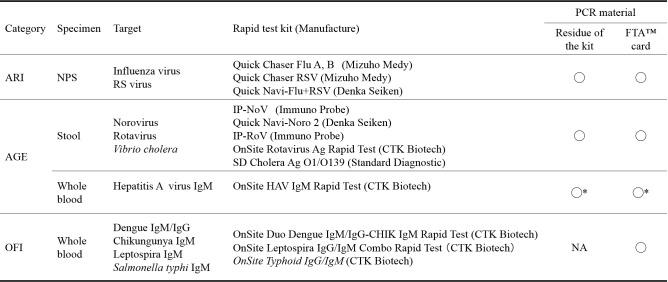
**Table 1. The list of the rapid test and materials for PCR used in this study.** ARI: acute respiratory infection, AGE: acute gastroenteritis, OFI, other febrile illness, RS: respiratory syncytial, NPS: Nasopharyngeal swab　NA: not applicable　*Stool samples were applied.

**Nucleic acid extraction**

Nucleic acids were extracted from residue samples used for the rapid test kits using QIAamp MinElute Virus Spin Kit, following the manufacturer’s protocol (QIAGEN, Hilden, Germany). For specimens applied on FTA^TM^ cards, punched discs were collected using a Harris Aluminum 1.2-mm Micro-Puncher (GE Healthcare Life Sciences, UK). To each tube with five discs per specimen, 150 µL of 1× PBS was added. Subsequent viral RNA extraction was performed using QIAamp Viral RNA Mini kit (Qiagen, Hilden, Germany), with modifications during the lysis stage. The sample in PBS was thoroughly mixed with 600 µL of Buffer AVL with carrier RNA (Qiagen, Hilden, Germany) and was incubated at room temperature for 10 minutes. The lysate was subjected to centrifugation at 13,000 rpm for 2 minutes to collect the disc debris at the bottom of the tube. Roughly 700 µL of the lysate was transferred to a new tube containing 560 µL of absolute ethanol. The succeeding steps for viral RNA extraction were performed according to the manufacturer’s protocol. The eluted viral RNA was subjected to complementary DNA (cDNA) synthesis.

**cDNA synthesis**

First-strand cDNA was synthesized using M-MLV Reverse Transcriptase (Invitrogen, Carlsbad, CA). The RT mix contained 50 mM Tris-HCl pH 8.3, 75 mM KCl, 3 mM MgCl2, 20 mM DTT, 2 mM dNTPs (0.5 mM each), 15 ng/µL random primers (Invitrogen, Carlsbad, CA), 1 U/µL RNaseOUT (Invitrogen, Carlsbad, CA), 5 U/µL units of M-MLV RT and 11 µL of RNA extract in a total reaction volume of 20 µL. The RT reaction profile was performed according to the manufacturer’s protocol. However, for AGE samples, the heat mixture step was changed into 98oC for 5 minutes and had additional DMSO.

**PCR assay**

TaqMan real-time PCR was used to detect influenza A virus^9^, influenza B virus^10^, respiratory syncytial virus (RSV)^11^, human metapneumovirus (hMPV)^12^, norovirus GI/GII^13^ and dengue virus^14^. Conventional PCR was used to detect entero/rhinoviruses^15^^,^ 16 and chikungunya virus^17^. Conventional nested PCR was used for rotavirus^18^ and hepatitis A virus^19^. The primers and target genes of each PCR assay were summarized in Supplemental table 1.

**Sequence analysis**

Sequencing was performed to identify the viruses detected as entero/rhinoviruses. PCR products were purified with QIAQuick PCR kit (Qiagen) and then analyzed using ABI 3730xl or ABI 3500 with BigDye version 1.1 (Applied Biosystems, Foster City, CA).

**Statistical analysis**

We performed McNemar’s test using R software (version 3.1.3, R Foundation for Statistical Computing, Vienna,) for comparison of the detection rate between two sampling materials, residue of samples used in rapid test kits and FTA^TM^ cards. Resulting *p*-value < 0.05 is considered as significant.

**Ethics Statement**

This study had been conducted as the outbreak surveillance after the calamity, which not requing IRB-RITM review. This had been conducted as the emergency response after the calamity. Identifier was not collected for purpose of the study, for purpose of clinical diagnosis. It was collected as a part of routine diagnosis. All samples were coded and stored without identifiers. Researcher had identifier to report the results to the hospital, which was part of the emergency response. We did not get consents from all of the patients since the samples were collected as a part of the clinical diagnosis using the rapid tests.

## Results

A total of 185 specimens were collected during the study period. Among them, 128 samples were collected from cases with ARI (69%), 17 samples from those with AGE (9.2%), and 40 samples from those with OFI (21.6%). Majority of ARI (120/128, 93.7%) and AGE (15/17, 88.2%) cases were children aged less than 5 years, while only 10% (4/40) of OFI cases were in this age group (Figure 3).

**Age distribution of the examined cases by main symptoms:**
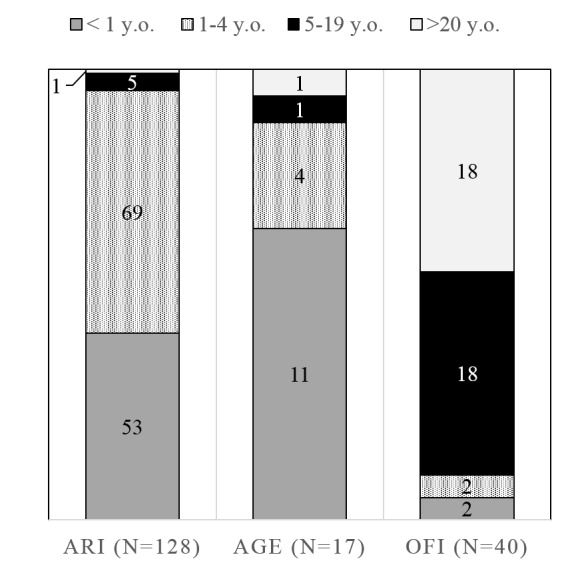

Rapid tests were conducted on site and the results are shown in Table 2 and 3. Eight (8) cases of influenza A and 2 cases of rotavirus were detected, while 2 cases were positive for dengue IgM. The summary of rapid test result and PCR using 2 types of samples (rapid test kit residues and FTA^TM^ cards) were shown in Table 2. While the rapid test could detect only Influenza A virus and rotavirus in ARI and AGE, respectively, more viruses were detected by PCR. For ARI cases, Influenza A virus, RS virus, hMPV, entero/rhinoviruses were detected. A significantly higher rate of entero/rhinoviruses was detected using the residue of rapid test buffer (n=46, 39.7%) than FTA^TM^ card (n=27, 21.4%) (Table 2). The species and serotype of 46 entero/rhinovirus positive samples were determined by sequencing, which identified rhinovirus A (n=11), rhinovirus B (n=5), rhinovirus C (n=8), and enterovirus D68 (EV-D68, n=12).

**Figure:**
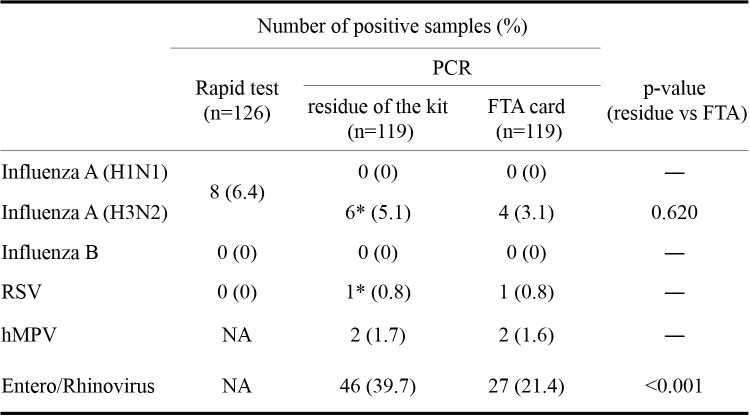
**Table 2. Comparison of the detection rate of ARI samples.** *one case was co-infection with influenza A and RS virus. ARI: Acute respiratory infections, RSV: respiratory syncytial virus, hMPV: human metapneumovirus, NA: not applicable.

For AGE samples, rotavirus and norovirus GII were detected from FTA^TM^ card, while only norovirus GII was detected from the residue of samples used in the rapid test kit (Table 3). Among 8 rotavirus positive samples, 3 were successfully genotyped by conventional PCR, which were all identified as G1P[8]. From blood samples, one dengue virus positive case and one chikungunya positive case were detected by PCR from FTA^TM^ card.

**Figure:**
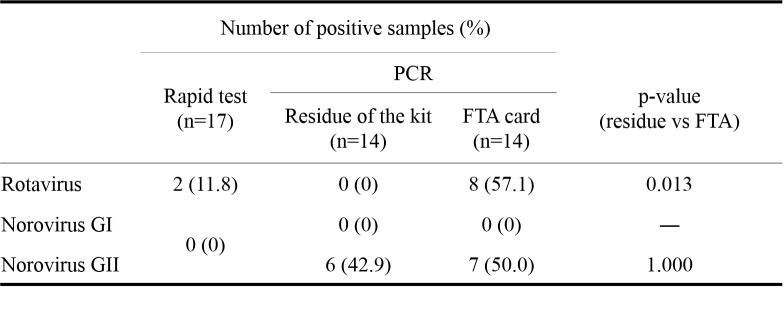
**Table 3: Comparison of the detection rate of AGE samples.** AGE: acute gastroentilitis

## Discussion

In general, during emergency settings such as post-disaster situations, available methods for laboratory confirmation of infectious diseases are limited. Commercially available rapid test kits are usually the only option for on-site diagnosis. However, since the variety of pathogens which could be detected by those kits is limited, it is not recommended to use only rapid test for disease surveillance unless there are particular pathogens to be identified. For example, among ARI samples, 6.4 % were positive by rapid test kit, while 48.1 % and 27.7 % were positive by PCR using the residue of rapid test kit and FTA^TM^ cards, respectively.

Thus, our study confirmed that the use of rapid test kits was not enough to detect the circulating infectious diseases in post-disaster setting, and the use of PCR-based assays should also be considered. The advantage of PCR is not only its ability to detect various pathogens, but also its amenability for further analysis such as subtyping or sequencing. Additionally, if the proper sample storage system is not available, aside from FTA^TM^ cards, even the residue of samples used for the rapid test could be utilized as PCR sample. Furthermore, the sample collection materials for PCR should be selected according to the type of specimen to be collected. Although FTA^TM^ card is known for its usefulness in field settings^20^, and the FTA^TM^ card is not always the best material as shown in our results. Although our study showed a high positivity rate of PCR from stool samples collected on FTA^TM^ cards, the positivity rate for NPS samples using FTA^TM^ cards was lower than the residue of rapid test kit. However, our sample size was not large enough to make any conclusions regarding the effectiveness of the FTA^TM^ card for NPS samples. For example, the result could be affected by the viscosity and the amount of applied specimen. Also, the storage condition should be considered. We have not verified the effect of storage temperature and its duration, so the detection rate might be different under appropriate cold chain. Also, the sensitivity of each PCR protocol should be considered as well, although we could not obtain nor calculate the detection limit of all the protocols used in this study. Although there are limited studies, which evaluated FTA^TM^ using NPS , one such report showed that the sensitivity of detection of avian influenza virus was reduced when the sample was applied to FTA^TM^ compared to direct examination of swab fluids^21^.

In our AGE samples, among 8 rotavirus positive samples, 3 samples were determined as G1P^8^, which was also reported as the predominant genotype in the Philippines^6^. The positivity rates of norovirus and rotavirus were concordant with our data from AGE cases under 5 years old (unpublished data). The distribution of enteroviruses revealed a high frequency of rhinoviruses and EV-D68. This result was in agreement with our previous study at EVRMC, that rhinoviruses were the most frequent viruses detected from severe pneumonia among children <5 years old^7^^,^ 22. Increased EV-D68 cases were also seen in this study. However, the impact of the typhoon on the circulation of EV-D68 is still unclear^23^.

In summary, our results suggest that PCR should be performed for infectious disease surveillance in post-disaster situations, and the materials which are not routinely used for PCR, such as residue of rapid test kit, could be utilized for detection. However, the positivity rate will be affected by several factors, e.g. the type of the specimen, amount of sample, sample collection materials, sensitivity of PCR. Further study is needed to determine the adequacy of collection tools and type of the specimen for PCR. In conclusion, our findings can be applied to infectious disease surveillance in a resource-limited setting which will contribute to management and control of disease outbreaks.

## Supporting Information

**Figure:** **Supplemental table 1:** Primers and probes used in this study Supplement table 1_Primer

**Figure:** **Supplemental table 2:** Cross table used for McNemar's test Supplement table 2_cross table

## Competing Interests

The authors have declared that no competing interests exist.

## Data Availability Statement

All relevant data are available within the paper.

## Corresponding Author

Hitoshi Oshitani (oshitanih@med.tohoku.ac.jp) and Mariko Saito-Obata (saitom@med.tohoku.ac.jp)
